# Tuning the endothelial response: differential release of exocytic cargos from Weibel‐Palade bodies

**DOI:** 10.1111/jth.14218

**Published:** 2018-08-12

**Authors:** T. D. Nightingale, J. J. McCormack, W. Grimes, C. Robinson, M. Lopes da Silva, I. J. White, A. Vaughan, L. P. Cramer, D. F. Cutler

**Affiliations:** ^1^ Centre for Microvascular Research William Harvey Research Institute Barts and the London School of Medicine and Dentistry Queen Mary University of London London UK; ^2^ MRC Laboratory of Molecular Cell Biology University College London London UK; ^3^ Imaging Informatics Division Bioinformatics Institute Singapore; ^4^ Department of Cell and Developmental Biology University College London UK

**Keywords:** exocytosis, hemostasis, inflammation, von Willebrand factor, Weibel‐Palade bodies

## Abstract

**Essentials**

- Endothelial activation initiates multiple processes, including hemostasis and inflammation.
- The molecules that contribute to these processes are co‐stored in secretory granules.
- How can the cells control release of granule content to allow differentiated responses?
- Selected agonists recruit an exocytosis‐linked actin ring to boost release of a subset of cargo.

**Summary:**

**Background**

Endothelial cells harbor specialized storage organelles, Weibel‐Palade bodies (WPBs). Exocytosis of WPB content into the vascular lumen initiates primary hemostasis, mediated by von Willebrand factor (VWF), and inflammation, mediated by several proteins including P‐selectin. During full fusion, secretion of this large hemostatic protein and smaller pro‐inflammatory proteins are thought to be inextricably linked.

**Objective**

To determine if secretagogue‐dependent differential release of WPB cargo occurs, and whether this is mediated by the formation of an actomyosin ring during exocytosis.

**Methods**

We used VWF string analysis, leukocyte rolling assays, ELISA, spinning disk confocal microscopy, high‐throughput confocal microscopy and inhibitor and siRNA treatments to demonstrate the existence of cellular machinery that allows differential release of WPB cargo proteins.

**Results**

Inhibition of the actomyosin ring differentially effects two processes regulated by WPB exocytosis; it perturbs VWF string formation but has no effect on leukocyte rolling. The efficiency of ring recruitment correlates with VWF release; the ratio of release of VWF to small cargoes decreases when ring recruitment is inhibited. The recruitment of the actin ring is time dependent (fusion events occurring directly after stimulation are less likely to initiate hemostasis than later events) and is activated by protein kinase C (PKC) isoforms.

**Conclusions**

Secretagogues differentially recruit the actomyosin ring, thus demonstrating one mechanism by which the prothrombotic effect of endothelial activation can be modulated. This potentially limits thrombosis whilst permitting a normal inflammatory response. These results have implications for the assessment of WPB fusion, cargo‐content release and the treatment of patients with von Willebrand disease.

## Introduction

A rapid response to vascular injury or infection minimizes blood loss and spread of pathogens. Endothelial rod‐shaped storage organelles called Weibel‐Palade bodies (WPBs) harbor multiple pre‐made, pro‐inflammatory and pro‐hemostatic proteins 1, 2, 3, including the leukocyte receptor P‐selectin, the pro‐hemostatic glycoprotein von Willebrand factor (VWF), pro‐inflammatory cytokines, and agents that control tonicity 4. Some cargos are up‐regulated after endothelial activation, including IL‐8 5, 6 and angiopoeitin‐2 7. Within minutes of secretagogue stimulation, WPBs undergo exocytosis 8, 9, releasing their content into the blood, which initiates hemostasis and leukocyte recruitment 2.

Release of VWF and P‐selectin have distinct functional consequences. VWF multimers are stored in multi‐concatamer coiled proteinacious tubules, together with their cleaved pro‐peptides 10. Upon exocytosis, the tubules unfurl into long protein strings, which recruit platelets even at non‐pathological shear 11, 12. VWF mutations or defective cellular machinery cause incorrect processing and can underlie bleeding disorders 13. Animal models of or patients with, low VWF exhibit a decreased incidence of atherosclerosis 14. Conversely, excess ultra‐high‐molecular‐weight VWF in the bloodstream (due to induced or genetic absence of the VWF‐cleaving metalloprotease ADAMTS‐13) results in the microvascular occlusions 15 of thrombotic thrombocytopenic purpura, and patients with elevated plasma VWF have an increased risk of cardiac events 14, 16 and stroke 17. VWF is thus a key factor in cardiovascular disease. P‐selectin is a leukocyte receptor that mediates initial rolling of leukocytes on the vascular endothelium 18, 19, 20. Loss or inappropriate clustering of P‐selectin at the endothelial cell surface results in immunodeficiency due to a failure to recruit leukocytes 20, 21.

Being co‐stored, parallel release at exocytosis of VWF and smaller components such as P‐selectin should be obligatory. However, there is evidence of differential release of VWF 22, 23, 24. At low extracellular pH, unfolding of VWF is prevented, such that only small soluble components are released 22, whereas in lingering kiss fusion, comprising about 10% of fusion events after strong histamine stimulation 23, only cargo proteins ≤40 kDa are released. However, neither of these mechanisms enables differential release of VWF vs. P‐selectin and therefore the segregation of inflammatory and hemostatic effects. Furthermore, no molecular machinery providing physiological control of VWF release has been identified.

Recent research has uncovered machinery controlling the efficiency of VWF release from WPBs 9, 25, 26. If differentially recruited by agonists, this would potentially promote regulated release of pro‐hemostatic VWF whilst not altering release of smaller pro‐inflammatory components. Such ‘differential release’, a novel layer of regulation, could limit potentially dangerous thrombosis whilst allowing a normal inflammatory response. We have used multiple *in vitro* assays to show that recruitment of an actomyosin ring allows differential release of cargo following stimulation by numerous physiologically relevant secretagogues. We also describe protein kinase‐C as upstream machinery that modulates its recruitment.

## Methods

### Cell culture and nucleofection

Human umbilical vein endothelial cells (HUVECs were cultured as described previously 27. GFP‐VWF 28 was from J. Voorberg and J.A. Van Mourik (Sanquin Research Laboratory, Amsterdam, the Netherlands). P‐sel.Lum–mCherry has been previously described 9. Lifeact‐GFP 29 was from B. Baum (University College London, London, UK). GFP‐tagged PKCα and PKCβ were gifts from A. Poole (University of Bristol, Bristol, UK). GFP‐tagged PKCδ and PKCε were from P. Parker (Francis Crick Institute, London, UK). DNA (1–5 μg) was nucleofected using program U‐001 (Lonza, Slough, UK). Cells were typically assayed 24 h post‐transfection.

### Immunofluorescence

This has been detailed previously 9.

### Secretion assay and ELISA

HUVECs were incubated with 1 μmol L^−1^ cytochalasin E (CCE) and 25 μmol L^−1^ blebbistatin (Sigma‐Aldrich, St Louis, MO, USA) for 5–15 min before determining VWF or pro‐peptide release in the presence or absence of 100 ng mL^−1^ phorbol 12‐myristate 13‐acetate (PMA) (Sigma‐Aldrich), 100 μmol L^−1^ histamine or 100 μmol L^−1^ histamine/10 μmol L^−1^ adrenalin/100 μmol L^−1^ 3‐isobutyl‐1‐methyl xanthine (IBMX) and/or the relevant drug for 30 min. VWF secretion assay and ELISAs have been described previously 30, 31. For VWF pro‐peptide secretion an ELISA kit (Mast Group Ltd, Bootle, Merseyside, UK) was used according to the manufacturer's instructions.

### Exocytic site labelling assay

Exocytic site labelling was performed using a modified method from Knop and Gerke 32. Confluent cells grown on 96‐well plates (Nunc, Roskilde, Denmark) for 2 days were washed in prewarmed release medium (M199 with 0.2% bovine serum albumin [BSA] and 10 mmol L^−1^ HEPES), and where necessary incubated with CCE or blebbistatin as for secretion assays. Cells were incubated for 2–20 min in the presence of rabbit anti‐VWF and either unstimulated or stimulated with phorbol 12‐myristate 13‐acetate (PMA) (6.25–100 ng mL^−1^), histamine (100 μmol L^−1^), thrombin (1 U μL^−1^), vascular endothelial growth factor (VEGF) (40 ng mL^−1^), Forskolin (10 μmol L^−1^), ATP (100 μmol L^−1^) or adrenalin (10 μmol L^−1^)/IBMX (100 μmol L^−1^), either alone or in combination, in release medium. Cells were incubated with wheat germ agglutinin (Thermo Fisher Scientific, Waltham, MA, USA) for 2 min on ice or fixed immediately in 4% paraformaldehyde, permeabilised with 0.2% Triton X‐100 in PBS and incubated with mouse anti‐VE‐cadherin (BD Biosciences, Franklin Lakes, NJ, USA) or with secondary antibodies conjugated to Alexa Fluor 488‐nm or 647‐nm and Hoescht 33342.

### High‐throughput image acquisition and segmentation

Cells were cultured, fixed and stained in 96‐well plates, then imaged with the Opera high‐content screening (PerkinElmer, Waltham, MA, USA) confocal microscope using a 40× air objective lens (NA 0.6). Datasets comprise 864 images (nine fields of view per well), approximately 10 000 cells. Analysis used Python2.7 with the scikit‐image library 33. Image noise was reduced by Gaussian blurring, then a binary mask was created using a threshold value from moment‐preserving thresholding 34. Adjacent sites were split using the marker‐based watershed flooding algorithm. Segmented objects beneath the resolution limit of the optical system were removed. Finally, morphometric measurements were taken. Segmentation was validated by comparison with a set of manually annotated images. Data analysis was conducted in the R programming language version 3.2 35.

### Western blotting

This was carried out as described previously 9.

### Live‐cell imaging

Nucleofected cells stimulated with PMA, histamine or histamine/adrenalin/IBMX were visualized as detailed previously using a 100× oil immersion lens (NA 1.4) on a spinning‐disk system (UltraVIEW VoX; PerkinElmer) 9.

### VWF string analysis and quantification

String assays were carried out as described previously 9.

### Rolling assay and quantification

HUVECs prepared as for string assays were placed on the stage of an Axiovert 200M microscope at 37°C, connected to a syringe pump, and Hanks Balanced Salts Solution (HBSS) perfused for 2 min at a constant wall shear stress 0.07 Pa (0.7 dyne cm^−3^). HUVECs were then either perfused with HBSS alone or stimulated with histamine/adrenaline/IBMX in the presence or absence of CCE (0.25 μmol L^−1^) for 5 min before being perfused with THP‐1 cells (0.5 × 10^6^/ml) in HBSS in the presence or absence of secretagogue. Videos were recorded using a Rolera Bolt CMOS camera (Ziosoft, Tokyo, Japan) using MicroManager software. Videos were analysed using ImageJ. Interacting THP‐1 cells were defined as those seen to pause on the endothelial monolayer and counted manually.

### Cell surface biotinylation assay

This was carried out as described previously 21.

## Results

**Figure 1:**
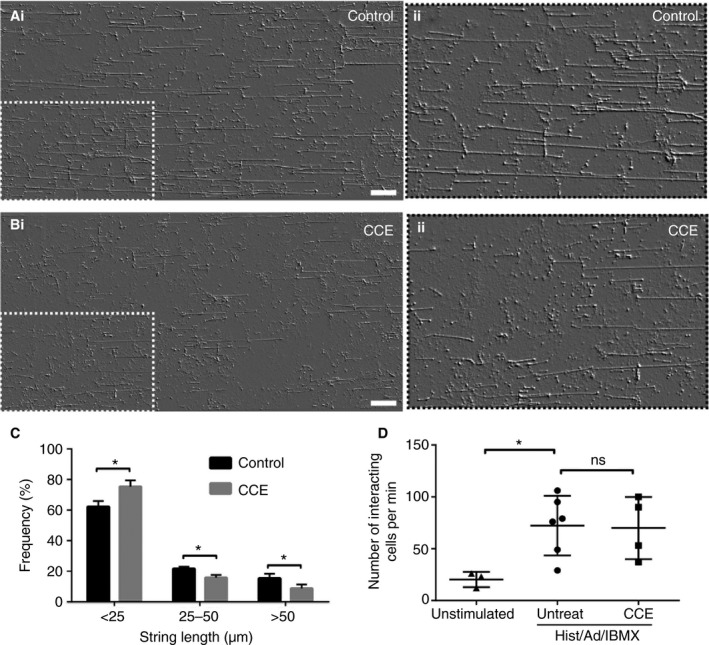
The actomyosin ring increases the efficiency of von Willebrand factor (VWF) string formation but has little effect on leukocyte rolling. Human umbilical vein endothelial cells (HUVECs) stimulated under flow with histamine (100 μmol L^−1^)/adrenalin (10 μmol L^−1^)/IBMX (100 μmol L^−1^) in the presence or absence of 0.25 μmol L^−1^ cytochalasin E (CCE) and fixed for string length analysis (A–C) or perfused with THP‐1 leukocytes for rolling analysis (D) (*n* = 3). (A, B) HUVECs were fixed and stained for VWF before imaging on a confocal microscope; pictures shown are tile scans of 10 fields of view. The whole image (i) and with the boxed area magnified (ii) are shown with a filter added to improve contrast. Scale bar 50 μm. (C) The lengths of VWF strings were quantified from three independent experiments (control, *n* = 13 images, 1346 strings; CCE, *n* = 14 images, 1364 strings). The percentage of strings less than 25 μm, between 25 and 50 μm and longer than 50 μm was calculated per image and standard error of the mean (SEM) shown. (D) The number of interacting THP‐1 leukocytes/min was determined from movies. Each point represents the total number of interacting leukocytes per 1‐min movie, with up to two movies acquired per experiment from stimulated cells. Error bars represent standard deviation (SD). Statistical significance assessed using the Mann–Whitney test (C) and one‐way anova with Dunnet's multiple comparison test (D). *P ≤ 0.05.

We and others demonstrated that VWF release is boosted by a contractile actomyosin ring forming around the fused WPB to squeeze out content 9, 25, 26, but whether this boost affects platelet and leukocyte recruitment to endothelial cells was undetermined. We first analyzed the effect of actomyosin ring inhibition on VWF string formation (Fig. 1A–C). HUVECs grown in flow chambers were briefly treated with a low dose (thus without an effect on cell viability or adherence) of the actin depolymerising drug CCE, which binds to the barbed end of the actin chain 36. A cocktail (to stimulate both calcium and cAMP‐mediated pathways of WPB exocytosis) of histamine, adrenalin and 3‐isobutyl‐1‐methyl xanthine (IBMX) was used to stimulate WPB exocytosis and the length of resulting strings was analyzed. In cells treated with CCE the formation of long strings was significantly reduced (Fig. 1A–C). To determine if leukocyte recruitment is also reduced we monitored rolling in the presence or absence of CCE. We saw no significant difference in the frequency of rolling leukocytes following drug treatment (Fig. 1D; Videos [Link], [Link], [Link]). Thus in a controlled system, inhibition of the actomyosin ring differentially affects inflammatory vs. hemostatic functioning. We hypothesize that the regulation of VWF secretion by the actomyosin ring is a secretagogue‐dependent way to bias the endothelial response to be more or less hemostatic.

**Figure 2:**
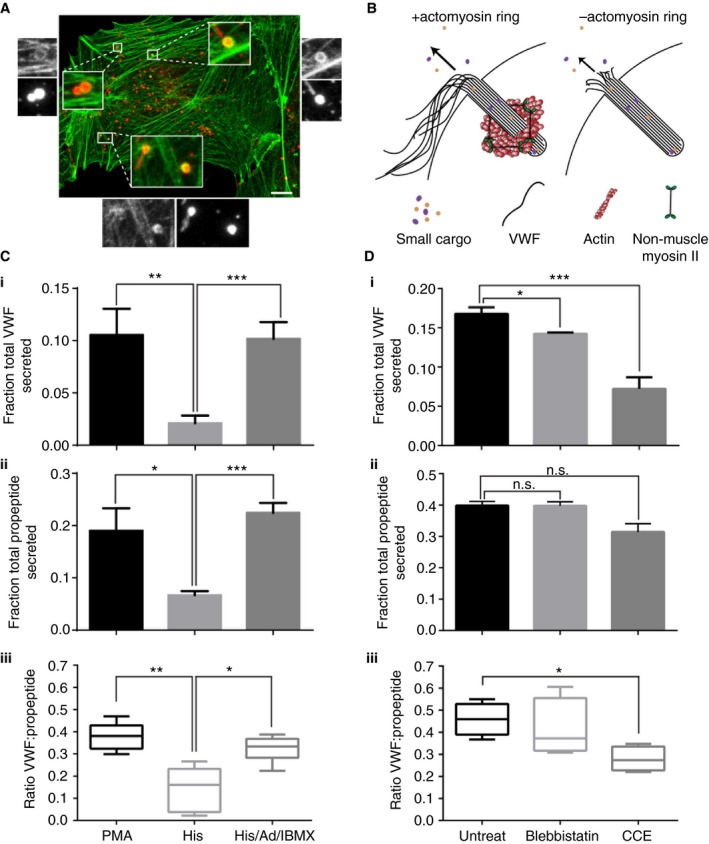
Different secretagogues release von Willebrand factor (VWF) and VWF pro‐peptide with differing efficiencies in a manner that is dependent on the actomyosin ring. (A) Human umbilical vein endothelial cells (HUVECs) were stimulated with 100 ng mL^−1^ phorbol 12‐myristate 13‐acetate (PMA) for 5 min and fixed using a procedure optimal for the actin cytoskeleton, co‐stained for VWF (red) and phalloidin (green) and imaged on a confocal microscope. Maximum intensity projections shown. Boxed regions are shown magnified. Bar 10 μm. (B) Schematic of Weibel‐Palade body exocytosis in the presence or absence of an actomyosin ring. Small cargo release is ring independent, whereas VWF release is more efficient in the presence of the ring. (C) Quantification of PMA (100 ng mL^−1^), histamine (100 μmol L^−1^) or histamine (100 μmol L^−1^)/adrenalin (10 μmol L^−1^)/3‐isobutyl‐1‐methyl xanthine (100 μmol L^−1^)‐stimulated (Ci) VWF or (Cii) pro‐peptide secretion (*n* = 6–9); error bars = SEM. (Ciii) Ratio of stimulated VWF:propeptide release. Boxes represent 25th–75th percentiles; whiskers represent minimum and maximum values. (D) Quantification of PMA (100 ng mL^−1^)‐stimulated (Di) VWF or (Dii) pro‐peptide secretion in the presence or absence of 25 μmol L^−1^ blebbistatin or 1 μmol L^−1^ cytochalasin E (*n* = 4); error bars = standard error of the mean (SEM). (Diii) Ratio of stimulated VWF:propeptide release. Error bars = SEM. Statistical significance assessed using *t*‐test with Welch's correction (Ci‐ii and Di‐ii) and ratio *t*‐test (Ciii and Diii). *P ≤ 0.05, **P ≤ 0.01 and ***P ≤ 0.001. [Color figure can be viewed at http://wileyonlinelibrary.com]

Von Willebrand factor is by far the largest WPB cargo protein and is likely to require the most physical force for efficient release, potentially explaining why control of the actin ring specifically affects hemostatic responses. Similarly, smaller pro‐inflammatory content should be less affected by actin ring inhibition (Fig. 2A,B). Further, to provide physiological regulation, different secretagogues should differentially utilize the actin ring. To test this we used three secretagogues that activate different downstream signaling pathways 4: PMA, histamine and histamine/adrenalin/IBMX. To determine the effect of content size on its efficiency of release, we compared secretion of VWF (large cargo) (Fig. 2Ci) with VWF pro‐peptide (small cargo) (Fig. 2Cii). The pro‐peptide is necessarily co‐packaged in equimolar amounts with VWF, thus providing exact ratiometric data. Further, the pro‐peptide is increasingly used clinically to determine VWF clearance 37, thus evidence of its differential release is of intrinsic interest. Although PMA and histamine/adrenalin/IBMX were similarly effective at exocytosing both large and small content, histamine releases the large cargo VWF much less efficiently than PMA. This was most clearly apparent when presented as the ratio of VWF/pro‐peptide release to give a measure of the secretion efficiency of large vs. small cargo (Fig. 2Ciii).

To determine if this difference in efficiency depends on the actomyosin machinery we used CCE to inhibit actin polymerization and blebbistatin 38 to block non‐muscle myosin II contraction (Fig. 2D). CCE completely inhibits ring formation, whereas blebbistatin reduces the rate of ring contraction. As predicted, efficient release of VWF following PMA stimulation requires the actomyosin ring; these inhibitors reduced VWF release (Fig. 2Di). Conversely, release of the smaller VWF pro‐peptide is essentially actin ring independent (Fig. 2Dii). The ratio of VWF to pro‐peptide release following PMA stimulation is 0.4 ± 0.05, whereas in cells that cannot recruit the ring, efficiency of release falls to 0.2 ± 0.04 (Fig. 2Diii). Interestingly, PMA‐stimulated cells in which the actin ring is inhibited behave similarly to histamine‐stimulated cells in terms of efficiency of VWF release. These data show that the actomyosin ring provides a means for secretagogue‐dependent control of VWF release without affecting smaller cargo.

**Figure 3:**
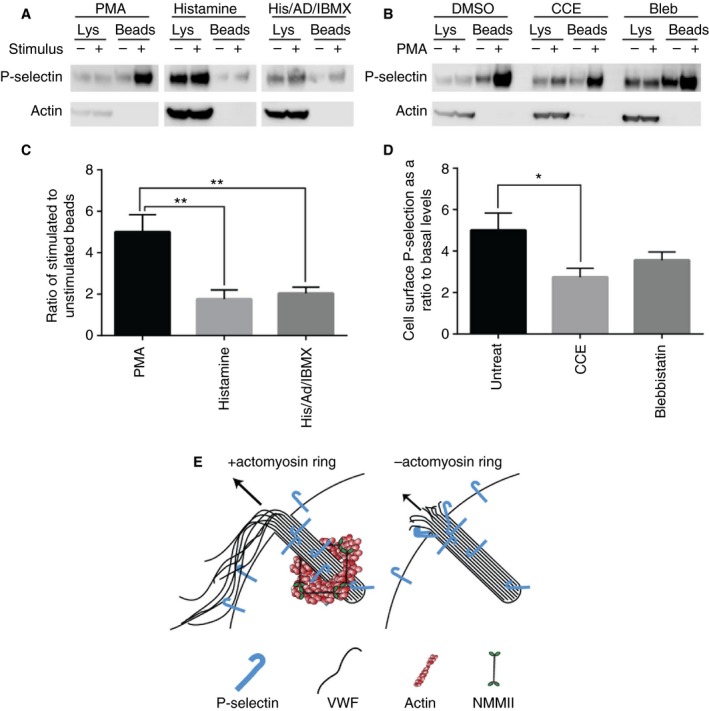
Release of P‐selectin from WPBs for recruitment to the plasma membrane is partially ring dependent. The proportion of cell surface to total P‐selectin levels was determined by surface biotinylation and neutravidin pulldown following stimulation with PMA (100 ng mL^−1^) (A, B), histamine (100 μmol L^−1^) or histamine (100 μmol L^−1^)/adrenalin (10 μmol L^−1^)/IBMX (100 μmol L^−1^) (A) or following PMA stimulation in the presence or absence of 25 μmol L^−1^ blebbistatin or 1 μmol L^−1^ CCE (B). Quantification of Western blots shown (C) PMA *n* = 11, his *n* = 3, HAI *n* = 6, (D) *n* = 12. (C, D) Error bars = standard error of the mean (SEM). Statistical significance assessed using *t*‐test with Welch's correction (C, D). *P ≤ 0.05, **P ≤ 0.01. (E) Schematic of WPB exocytosis in the presence or absence of an actomyosin ring. WPB, Weibel‐Palade body; NMMII, non‐muscle myosin II. [Color figure can be viewed at http://wileyonlinelibrary.com]

We next addressed whether the actomyosin ring influenced the delivery of integral membrane proteins to the cell surface from WPBs. We stimulated HUVECs with different secretagogues and monitored P‐selectin appearance on the plasma membrane (Fig. 3A,C). The most efficient delivery to the plasma membrane occurred following PMA stimulation, whereas histamine and histamine/adrenalin/IBMX behave similarly. The delivery of P‐selectin to the cell surface in response to PMA was partially dependent on the actomyosin ring as both blebbistatin and CCE reduced cell surface levels (Fig. 3B,D). The reason for this is unclear but might reflect VWF/P‐selectin binding, retaining P‐selectin within the WPB after fusion 39. Consistent with this, as reported, P‐selectin is enriched along VWF strings 40 and at exocytic sites post‐exocytosis (Figure S1 and Fig. 3E). Alternatively, the partial inhibition could reflect a steric hindrance of the extracellular domain of P‐selectin as it exits the fusion pore; P‐selectin mobility is limited in mature WPBs 41. Therefore, the delivery of larger integral membrane proteins can be influenced by inhibition of the actin ring, although not enough to inhibit function (Fig. 1D).

**Figure 4:**
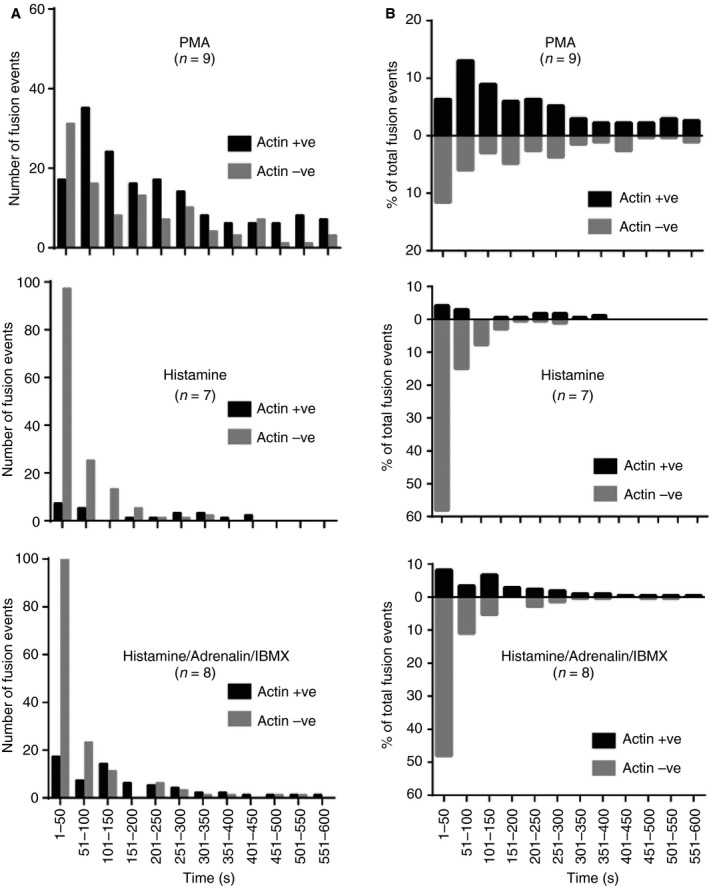
Actin ring recruitment is secretagogue and time dependent. Human umbilical vein endothelial cells (HUVECs) were nucleofected with mCherry‐PselectinLum domain and lifeactGFP and imaged with a spinning‐disk confocal microscope in the presence of 100 ng mL^−1^ PMA (*n* = 9), 100 μmol L^−1^ histamine (*n* = 7) or 100 μmol L^−1^ histamine/10 μmol L^−1^ adrenalin/100 μmol L^−1^ IBMX (*n* = 8). Z stacks were acquired at a spacing of 0.5 μm every 5 s for 10 min. (A) The frequency of fusion events with (positive +ve) or without an actin ring (negative −ve) at each time‐point is plotted. (B) The percentage of actin ring‐positive (+ve) or negative events (−ve) compared with the total number of events is plotted.

To directly determine the extent and kinetics of actin ring recruitment, we monitored actin ring recruitment in live cells 9. We monitored the loss of mcherry‐Pselectin.lum (marking WPB fusion) and the recruitment of lifeact‐GFP (tracking ring assembly) and found (Fig. 4A) that approximately ≈15% of histamine‐stimulated fusion events, ≈40% of histamine/adrenalin/IBMX‐stimulated events and ≈65% of PMA‐stimulated events recruit the ring. Additionally, the probability of ring recruitment increases over time (Fig. 4B). Immediately following stimulation (0–50 s), and irrespective of secretagogue, the likelihood of recruitment is low. For PMA and histamine/adrenalin/IBMX‐stimulated cells this is followed by increased ring recruitment (50–200 s) (68% and 69% of events are actin‐positive in PMA and histamine/adrenalin/IBMX‐stimulated cells, respectively, over 100–600 s). In histamine‐stimulated cells the majority of events are actomyosin ring independent, although the percentage of actin‐positive events increases over time, until every event recruited a ring (although few events occur at these later times). Therefore time‐dependent phenomena are likely to be required for ring recruitment, presumably including both signaling and recruitment of machinery.

**Figure 5:**
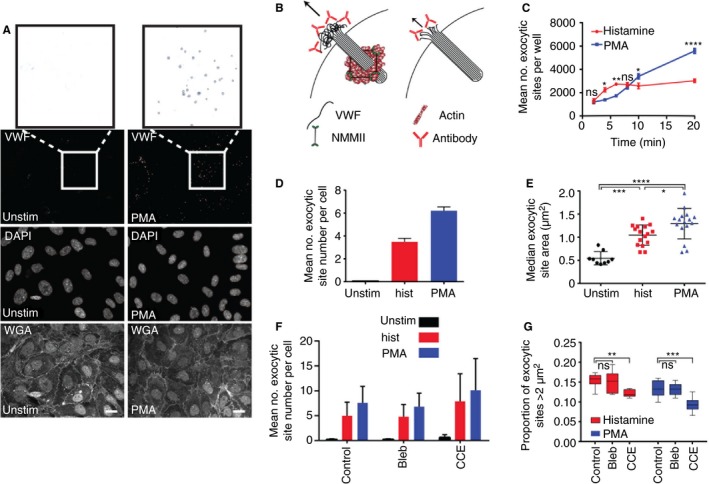
High‐throughput analysis of exocytic events. (A) Human umbilical vein endothelial cells (HUVECs) were unstimulated or stimulated with 100 ng μL^−1^ PMA for 10 min, followed by staining for external von Willebrand factor (VWF), plasma membrane with wheat germ agglutinin (WGA) and the nucleus (DAPI, 4′,6‐diamidino‐2‐phenylindole). Nine fields of view were acquired per well and eight wells imaged per condition. External VWF was segmented using a custom‐designed program. Boxed areas on the VWF channel are shown inverted and at higher magnification as examples of segmented sites typically acquired from unstimulated and PMA‐stimulated cells. Scale bar 20 μm. (B) Schematic of external antibody labelling protocol to differentiate between actomyosin‐dependent and independent exocytosis. NMMII, non‐muscle myosin II. (C) HUVECs stimulated with either histamine (100 μmol L^−1^) or PMA (100 ng mL^−1^) were fixed following 2–20 min of stimulation. The number of segmented external exocytic sites was calculated for each well (the sum of nine fields of view) for each time‐point and mean and standard error plotted (*n* = 8 wells). A representative experiment is shown from *n* = 4 independent experiments. (D and E) HUVECs were stimulated for 10 min with PMA (100 ng mL^−1^) or histamine (100 μmol L^−1^) or left unstimulated. The mean number of exocytic sites per cell per well (D) (*n* = 8 wells, a representative experiment is shown from *n* = 3 independent experiments) and the median area per site (E) (*n* = 9–16 independent experiments) are shown. Bars represent standard error of the mean (SEM). (F and G) HUVECs were untreated or pretreated with blebbistatin (25 μmol L^−1^) or cytochalasin E (CCE) (1 μmol L^−1^) for 15 min before stimulation with histamine and PMA. The mean number of sites per cell (F) (*n* = 3 independent experiments) and the proportion of sites with area greater than 2 μm^2^ (G) (*n* = 8 wells, a representative experiment from *n* = 3 independent experiments is shown). Boxes represent 25th–75th percentiles; whiskers represent minimum and maximum values. Statistical significance was assessed using two‐way anova with Sidak's multiple comparison test (C and G), or one‐way anova with Tukey's multiple comparison test (E). *P ≤ 0.05, **P ≤ 0.01, ***P ≤ 0.001, ****P ≤ 0.0001. [Color figure can be viewed at http://wileyonlinelibrary.com]

We next sought to determine whether recruitment of the actomyosin ring following stimulation by the many established WPB secretagogues 4, 42, both alone and in combination, is a major feature of exocytosis. We developed an assay for determination of the size of the fusion site. This assay takes advantage of information obtained previously using correlative light and electron microscopy and scanning electron microscopy showing that levels of exocytosed, antibody‐accessible VWF are dependent on the actomyosin ring 9. We added anti‐VWF antibody to the media to retain VWF at exocytic sites (and prevent string formation) 32, 43 to analyze exocytic site formation in thousands of cells. We hypothesized that more efficient release mediated by the actin ring is likely to result in bigger sites (Fig. 5B), and developed an automated segmentation protocol to acquire a set of morphological measurements for each site from 72 fields of view (950–1200 cells) analyzed per condition (Fig. 5A).

This approach is unbiased, automated and highly sensitive, as shown by our analyses revealing that the number of sites increased in response to increased PMA in a dose‐dependent manner (Figure S2Aa), without change to the site area (Figure S2Ab). Thus, even at a high density, segmentation of individual sites is not compromised. Histamine elicits a rapid response, typically complete by 10 min post‐stimulation, whereas PMA produces a more linear release of VWF 44. Importantly, these biochemical dynamics were replicated in our assay, which is sensitive enough to distinguish differences in the number of exocytic sites over discrete 2‐min periods (Fig. 5C). To verify the assay could differentiate ring‐dependent and independent exocytic events, we monitored the number (Fig. 5D) and area of sites (Fig. 5E) and noted that sites segmented from stimulated cells were significantly larger than those from unstimulated cells, and that PMA‐stimulated cells produce larger sites than histamine‐stimulated cells, correlating with actin ring recruitment. We therefore analyzed changes in histamine and PMA‐stimulated cells treated with blebbistatin and CCE to determine the effect of actin ring inhibition on the proportion of large exocytic sites (classified as those greater than 2 μm^2^) (Fig. 5G and Figure S2B). CCE treatment specifically reduces the proportion of larger exocytic sites following both PMA and histamine stimulation, whereas blebbistatin, as expected, had little effect (as it slows rather than completely inhibits actomyosin ring contraction). This effect was greatest in PMA‐stimulated cells (Fig. 5G and Figure S2B). Together these results validate a new, sensitive, high‐throughput method of monitoring VWF exocytic sites suitable for screening secretagogues for their ability to recruit the actomyosin ring.

**Figure 6:**
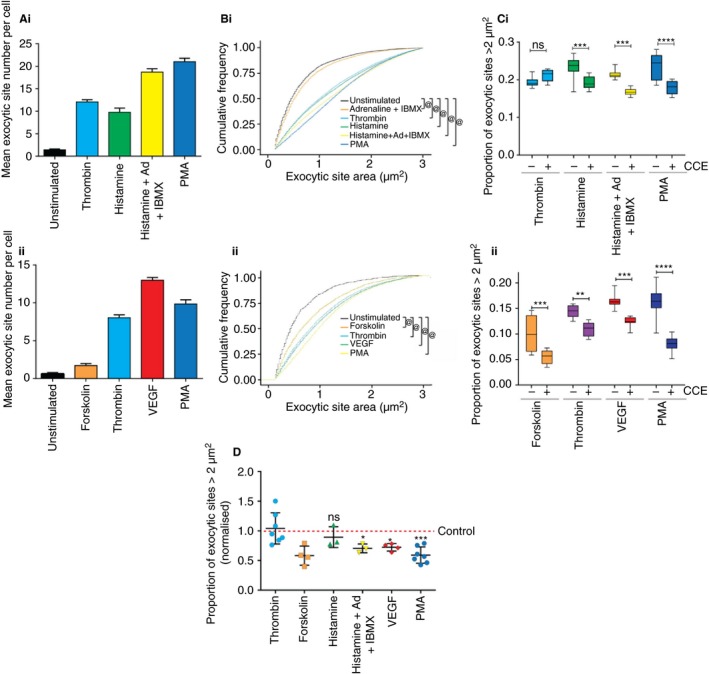
Analysis of actin ring function with a variety of secretagogues. Human umbilical vein endothelial cells (HUVECs) were treated with or without 1 μmol L^−1^ cytochalasin E (CCE) before being stimulated with 100 ng μL^−1^ PMA, 100 μmol L^−1^ histamine, 1 U mL^−1^ thrombin, 10 μmol L^−1^ adrenalin/100 μmol L^−1^ IBMX, 100 μmol L^−1^ histamine/10 μmol L^−1^ adrenalin/100 μmol L^−1^ IBMX, 10 μm forskolin/100 μmol L^−1^ IBMX, or 40 ng mL^−1^ vascular endothelial growth factor (VEGF) for 10 min, followed by staining for external von Willebrand factor (VWF) and the nucleus. Nine fields of view were acquired per well, and eight wells imaged per condition. Data from representative experiments shown (A–C) (*n* = 3) and the mean of three to seven experiments (D). (A) Mean number of exocytic sites per cell per well following secretagogue stimulation. Bars are SEM (*n* = 8 wells). (B) Cumulative frequency graph shows the distributions of the area of exocytic sites. (C) The mean proportion of exocytic VWF‐positive sites with area greater than 2 μm^2^ was calculated following stimulation with various secretagogues with and without CCE (1 μmol L^−1^). Boxes represent 25th–75th percentiles; whiskers represent minimum and maximum values. *n* = 8 wells. (D) The mean proportion of exocytic sites with area greater than 2 μm^2^ following stimulation with a number of secretagogues in the presence of CCE normalized to the mean proportion of large sites in control samples. Mean value is derived from the *n* = 8 wells per experiment (*n* = 3–7). Statistical significance assessed between stimulated and unstimulated distributions using a two‐sample Kolmogorov–Smirnov test (B), two‐way anova with Sidak's multiple comparison test (C) and one‐way anova with Dunnet's multiple comparisons test (D). *P ≤ 0.05, **P ≤ 0.005, ***P ≤ 0.001, ****P ≤ 0.0001, ^@^ *P* ≤ 10^−15^. [Color figure can be viewed at http://wileyonlinelibrary.com]

We then surveyed different secretagogues for their use of the actomyosin ring. By determining the number (Fig. 6A and Figure S3A,B) and size of exocytic sites (Fig. 6B–D and Figure S3C,D) following stimulation with different secretagogues in isolation or combination, we found significant differences in actin ring recruitment (Fig. 6C,D, and Figure S3C–F). Although thrombin relies minimally on the actin ring for release of VWF, PMA, VEGF, histamine/adrenalin/IBMX and forskolin are strong ring recruiters (correlating with our and others’ findings 9, 25). We also find that actin ring recruitment can be enhanced via addition of some, but not all, calcium or cAMP‐raising agents (Fig. 6 and Figure S3), which is consistent with our earlier ELISA data. Our approach provides large quantitative datasets to reveal actin ring‐dependence for a range of secretagogues and indicates for the first time that endothelial cells, by responding to physiological cues, have the capability to tune the release of cargo content.

We next sought the upstream machinery required for actin ring recruitment. Actin‐dependent exocytic structures occur in the cortical granules of *Xenopus* oocytes, the zymogen granules of the pancreatic and parotid acinar and the lamellar bodies of type II pneumocytes 45. Some of these granules utilize protein kinase C (PKC) isoforms to recruit an actin ring 46, 47. Given this, and that PMA (an activator of classical and non‐classical PKC isoforms) recruits the actin ring most efficiently, we analyzed the role of PKC in ring recruitment.

**Figure 7:**
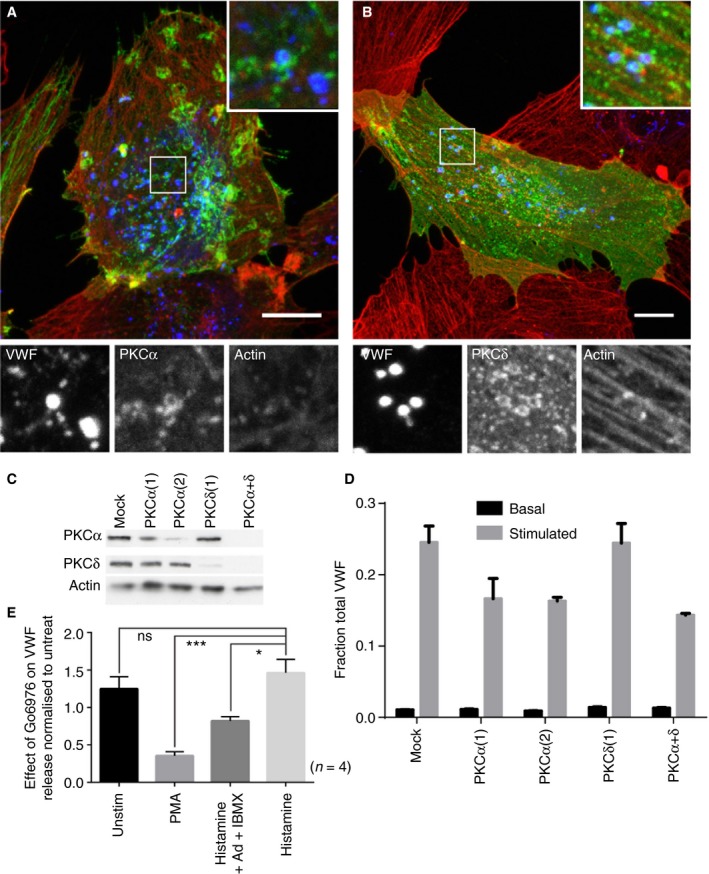
The role of protein kinase C isoforms in actin ring recruitment and von Willebrand factor (VWF) secretion. (A, B) Human umbilical vein endothelial cells (HUVECs) were nucleofected with PKCαGFP (A) or PKCδGFP (B) and stimulated for 5 min with 100 ng mL^−1^ PMA, fixed in formaldehyde with a procedure optimal for the actin cytoskeleton, co‐stained for VWF (blue) and phalloidin (red) and imaged on a confocal microscope. Image shown is a maximum intensity projection; boxed regions are shown magnified. Bar 10 μm. (C, D) HUVECs were nucleofected with two rounds of 200 pmol siRNA against PKCα, δ or both isoforms together and either (C) the samples were prepared for Western blot or (D) VWF secretion monitored. (E) HUVECs were treated with 1 μmol L^−1^ GÖ6976 and then stimulated with PMA (100 ng mL^−1^), histamine (100 μmol L^−1^) or a combination of histamine (100 μmol L^−1^), adrenalin (10 μmol L^−1^) and IBMX (100 μmol L^−1^). VWF secretion was monitored and results are shown normalized to the uninhibited sample. The protein kinase C (PKC) inhibitor has the greatest effect on PMA‐stimulated release and a lesser effect on hist/ad/IBMX. *n* = 4, error bars = standard error of the mean (SEM). Statistical significance assessed using *t*‐test with Welch's correction. *P < 0.05 and **P < 0.01. [Color figure can be viewed at http://wileyonlinelibrary.com]

PKCα, PKCδ, PKCε, PKCη and PKCζ are expressed in HUVECs 48. Live‐cell imaging of individual fusion events using mCherry‐P‐selectinLum as a marker of fusion and various human GFP‐tagged versions of PKC showed recruitment of PKCα and PKCδ on the actin ring (Fig. 7A,B) but not epsilon or beta (not endogenously expressed; data not shown), suggesting specific recruitment. Recruitment of PKC prior to the actin ring is consistent with a role upstream of or during initiation of ring recruitment (Figure S4). To assess the function of these isoforms in VWF release from PMA‐stimulated cells we depleted either PKCα or PKCδ (Fig. 7C) and monitored VWF release by ELISA. PKCα but not PKCδ knockdown had a marked effect (Fig. 7D).

Finally, we monitored the effect of an inhibitor of PKCα on VWF secretion (Fig. 7E). Predictably, PKCα inhibition had the strongest effect on PMA‐stimulated release, a lesser effect on histamine/adrenalin/IBMX‐stimulated release and no effect on histamine‐stimulated release (Fig. 7E and Figure S5A). We also noted some reduction in the number of exocytic sites seen in PMA‐stimulated cells, with a lesser effect on histamine or histamine/adrenalin/IBMX (Figure S5B,C). An additional role for PKC in exocytosis is thus possible alongside the formation of the actomyosin ring.

## Discussion

We present here evidence for the differential release of WPB cargo that we speculate can allow the separation of hemostatic and inflammatory responses. We find that different secretagogues are differentially effective at recruiting an actomyosin ring to WPBs at exocytosis, and that recruitment of this ring correlates with the release of the largest WPB cargo protein, VWF. Ultimately this represents a new layer of control to facilitate greater regulation over the outcome of endothelial activation.

We firstly demonstrated that two functions ascribed to WPB cargo content can be differentially regulated. We used an *in vitro* flow chamber to separate effects on leukocyte adhesion from VWF string formation in endothelial cells treated with a low dose of the actin poison CCE. Recruitment of the actomyosin ring affected VWF string formation (and therefore the efficiency of platelet recruitment) (Fig. 1A–C) but not leukocyte recruitment (Fig. 1D).

To determine if this effect reflects size‐specific control of the release of WPB cargo, we monitored release of equimolar co‐packaged VWF multimers (large protein) vs. VWF pro‐peptide (small protein) in parallel. We found that only the release of VWF was differentially evoked by secretagogues, and that this correlated with the recruitment of an actin ring (Fig. 2). Stimulation with histamine alone was not efficient at releasing VWF relative to the pro‐peptide, whereas PMA or histamine/adrenalin/IBMX were much more efficient (Fig. 2C). Similar reductions in efficiency followed perturbation of actomyosin ring function at PMA stimulation plus blebbistatin or CCE (Fig. 2D); squeezing by the actin ring is more important for larger cargoes than small ones, and this can explain some of the differences revealed by functional assays. The greater effects of CCE than blebbistatin probably reflect the fact that CCE inhibits ring formation whereas blebbistatin only slows the rate of its contraction.

Leukocyte rolling following secretagogue stimulation is initiated by P‐selectin 19, 20 clustered at the cell surface by the WPB co‐cargo CD63 21. CD63 readily transfers to the plasma membrane even in situations where VWF release is inhibited, including release at low pH 22 or during lingering kiss fusion 23. Surface biotinylation demonstrated that P‐selectin traffic to the cell surface is partially actomyosin ring‐dependent (Fig. 3), although we see no difference in leukocyte rolling following actomyosin ring inhibition (Fig. 1D). Direct interactions with VWF (also suggested by imaging, Figure S1) may explain this effect 39. The clustering effects of CD63 or a simple excess of receptor may help to mitigate the differences seen in P‐selectin recruitment to the cell surface.

Directly imaging ring recruitment (Fig. 4) to determine which secretagogues recruit the ring to the greatest extent corroborated our ELISA results. Notably, we also identified a time dependence for ring recruitment, with later exocytic events with all tested stimuli much more likely to recruit the ring. This intriguing time course suggests that downstream signaling is required both to recruit the actin ring and to localize associated cellular machinery.

The most efficient actin ring recruitment (and therefore VWF release) occurs when multiple secretagogues are used (Figs. 2 and 4). We utilized a new high‐throughput approach to monitor a range of secretagogues (Figs 5, 6 and Figure S2). Assaying thousands of exocytic sites from thousands of cells, this approach affords excellent temporal sensitivity and statistical significance. *In vivo* the endothelium is likely to be stimulated by multiple secretagogues; histamine activation is accompanied by at least some adrenalin (resting levels are 0.31 nmol L^−1^ 49). Identifying the signaling pathways downstream of secretagogue activation is complex, as many intersect. One of the strongest ring‐promoting agents is the non‐physiological Diacylglycerol (DAG) analogue PMA, suggesting PKC involvement (most likely PKCα) (Fig. 7). Because PKCα can be activated directly by both DAG and calcium 50 or indirectly via cAMP‐dependent agonists and EPAC (exchange proteins directly activated by cAMP isoform) 51, 52, PKC activation could feasibly occur downstream of any of the ring‐recruiting agonists that we, and others, have identified. The cAMP‐raising secretagogues forskolin and adrenalin have previously been identified by others 25 as stimulating actin ring recruitment. Here we find that addition of adrenalin/IBMX to the calcium‐dependent secretagogue histamine significantly enhances ring recruitment, suggesting that activation of cAMP may be an important route to ring recruitment. Interestingly, thrombin, which is largely actin ring independent, can inhibit cAMP production, potentially explaining why this is a poor ring recruiter 53. However, it is likely that other, as yet unidentified, pathways independent of PKCα are also involved in ring recruitment. Histamine alone is able to recruit the actin ring, despite the fact that its mode of action is thought to be PKC independent 48. Although PKCα acts in ring recruitment, VEGF is also effective at recruiting the actin ring and acts via PKCδ (and VWF release is not inhibited by inhibition of PKCα in VEGF‐stimulated cells) 48, thus roles for additional PKC isoforms are possible.

Von Willebrand factor release is not completely actin ring dependent, as CCE treatment does not abolish it, and secretagogues that do not utilize the ring still expel VWF, albeit at a lower efficiency (Figs. 2, 3, 4, 5, 6), as measured by pro‐peptide vs. VWF release. Thus, cargo expulsion may also be driven by water entry, changes in ionic fluxes or pH 54. We have also found protracted actin ring formation and slower release of content at lowered external pH (data not shown). Other large acidified granules with viscous content including lamellar bodies, and pancreatic and parotid acinar zymogen granules all require extra machinery to drive release 45, indicating that charge is not always the sole and most efficient driving force. Other large granules may also exhibit differential recruitment of actin rings and therefore differential release of content.

Our research complements recent research confirming that VWF release is boosted by an actomyosin ring 25, 26. However, there are differences in the findings. We concluded that rings form *de novo* after fusion 9, whereas Han *et al*. report actin remodeling before fusion from a pre‐existing framework. We also differ on whether WPB localization is generally affected by myosin II inhibition 9, 26. The different conclusions might reflect which cell surface was imaged (apical vs. basal) or spinning disk vs. custom microscopy 9, 25. A role for some actin nucleation remains a possibility.

Differential release has previously been proposed, based on the presence of multiple pools of WPBs 24. Although additional cargos can be added to WPBs, including IL‐8 5, 6 and angiopoeitin‐2 7, we have no evidence to suggest the ring can be differentially recruited to distinct WPBs containing different cargos; this would require cytoplasmic machinery detecting cargo stored internally in WPBs.

Our results support the clinical use of VWF pro‐peptide monitoring, perhaps immediately after agonist treatment, where needed. We also note that DDAVP, the secretagogue most commonly used to treat VWD patients, is cAMP‐dependent 55 and perhaps this is one reason why it is an effective therapeutic choice.

In conclusion, these data provide evidence for an additional level of functional control of WPBs, concluding that endothelial cells may tune the hemostatic response via the recruitment of an actomyosin ring.

## Addendum

J. J. McCormack and T. D. Nightingale made the most substantial contributions to this paper. J. J. McCormack carried out quantification and statistical analysis of images, invented novel assays, designed and carried out experiments, analyzed data and wrote the manuscript. T. D. Nightingale invented novel assays, designed and carried out experiments, analyzed data and wrote the manuscript. C. Robinson, W. Grimes and M. Lopes da Silva designed and carried out experiments and contributed to the writing of this paper, I. J. White and A. Vaughan provided technical expertise and analyzed data, and L.P. Cramer and D.F. Cutler designed research, analyzed data and wrote the paper.

## Disclosure of Conflict of Interests

T. D. Nightingale was funded by a Medical Research Council project grant MR/M019179/1 and a British Heart Foundation project grant (PG/15/72/31732). J. J. McCormack and D. F. Cutler were funded by an MRC programme grant MC_UU_12018/2. I.J. White was funded by MRC LMCB core. W. Grimes was supported partly by the Biomedical Research Council of A*STAR (Agency for Science, Technology and Research), Singapore, partly by the MRC LMCB. C. Robinson was funded by a BHF project grant (PG/15/72/31732). M. Lopes da Silva reports grants from MRC during the conduct of the study, and grants from MRC outside the submitted work. The other authors state that they have no conflict of interest.

## Supporting information


**Fig. S1.** P‐selectin localization at exocytosis.
**Fig. S2.** High‐throughput analysis of exocytic events.
**Fig. S3.** Analysis of actin ring function with a variety of secretagogues.
**Fig. S4.** The timing of PKC delta recruitment to exocytic sites.
**Fig. S5.** Effect of GÖ6976 on exit site size and number.

**Video S1.** Rolling analysis of untreated endothelial cells.

**Video S2.** Rolling analysis of endothelial cells stimulated with histamine and adrenalin.

**Video S3.** Rolling analysis of untreated endothelial cells.
