# Supplementary material for: Tuning the endothelial response: differential release of exocytic cargos from Weibel‐Palade bodies
Source: J Thromb Haemost. 2018 Aug 12;16(9):1873–86. doi: 10.1111/jth.14218 (PMC6166140; doi:10.1111/jth.14218)
Supplement: Supplementary file 1 — Fig. S1. P‐selectin localization at exocytosis. Fig. S2. High‐throughput analysis of exocytic events. Fig. S3. Analysis of actin ring function with a variety of secretagogues. Fig. S4. The timing of PKC delta recruitment to exocytic sites. Fig. S5. Effect of GÖ6976 on exit site size and number. [file JTH-16-1873-s001.docx]

Tuning the endothelial response: differential release of exocytic cargos from Weibel-Palade Bodies.

**Supplementary Material**


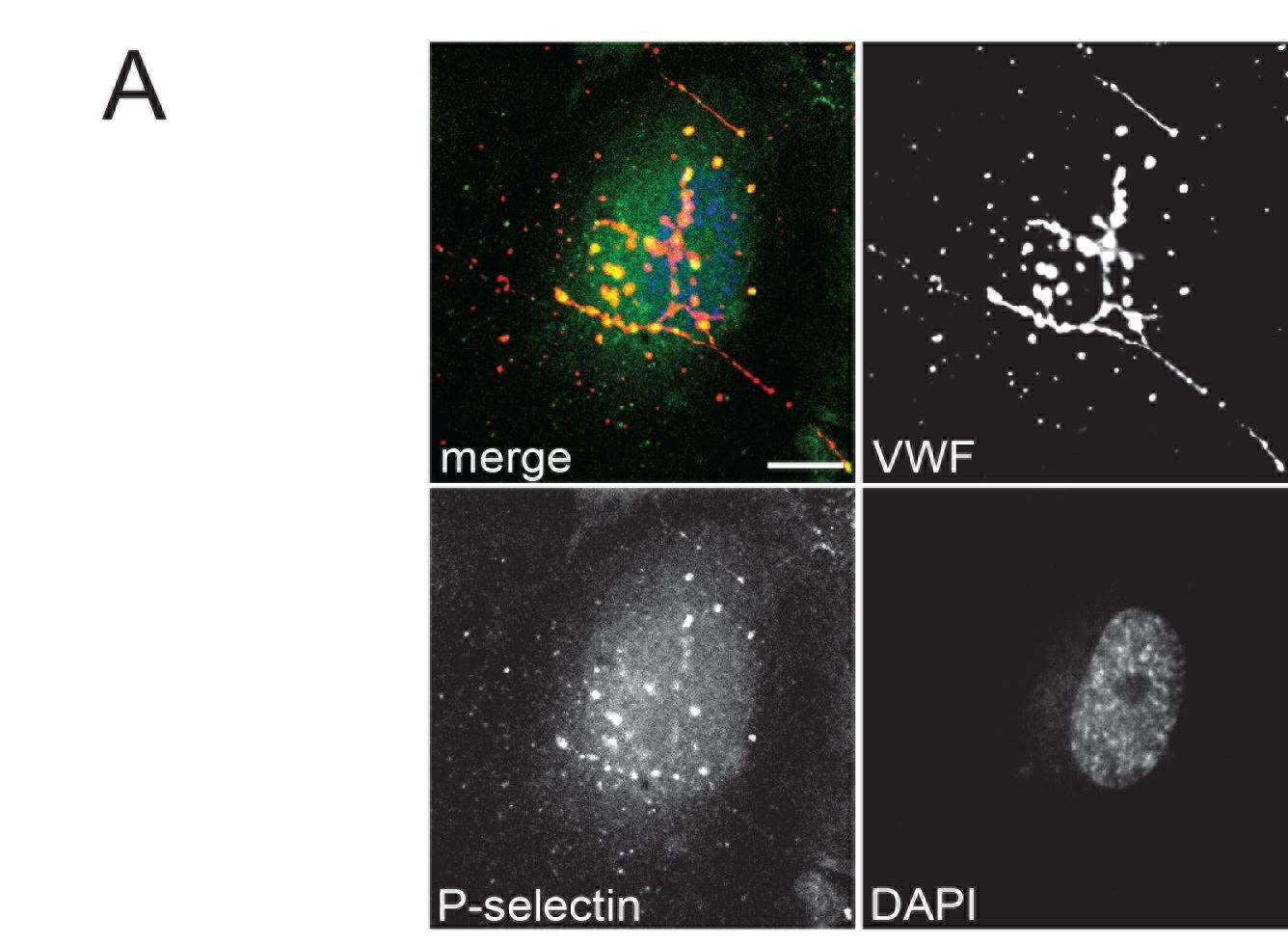


Figure S1. **P-selectin localisation at exocytosis**

HUVECs were stimulated with 100 ng/ml PMA for 20 min and labelled without permeabilisation for surface VWF (red), P-selectin (green), alongside the nucleus (blue) and imaged on a confocal microscope. Images shown are maximum intensity projections. Bar 10 µm.


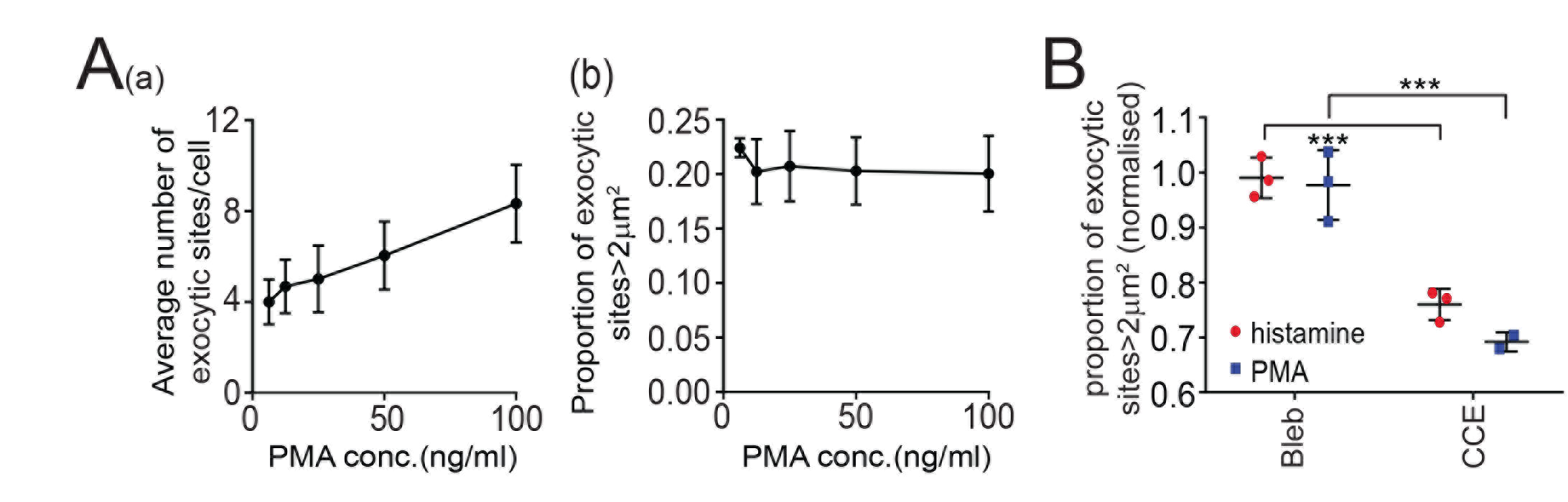


Figure S2 **High-throughput analysis of exocytic events.**

HUVECs were stimulated with 100ng/ml PMA for 10 minutes, or left unstimulated followed by staining for external VWF, plasma membrane with wheat germ agglutinin (WGA) and the nucleus (DAPI). Images were acquired using the Opera high-content screening (PerkinElmer) confocal microscope. Nine fields of view were acquired per well, and eight wells imaged per condition. External VWF was segmented using a custom-designed program. (A) HUVECs were stimulated with serial dilutions of PMA (100ng/ml – 6.25ng/ml) for 10 minutes and the number and area of segmented external exocytic sites measured. The average number of exocytic sites per cell (Aa) and average proportion of exocytic sites with area greater than 2µm^2^ (Ab) is shown. Bar represent SEM (n=4). Statistical significance was assessed using 1-way ANOVA with Tukey’s multiple comparison test (Ab) and no significant difference is seen between any concentration of PMA. (B) HUVEC were pre-treated with blebbistatin (25 μM) or CCE (1 μM) for 15 min before stimulation with histamine and PMA. The mean proportion of larger (area greater than 2µm^2^) exocytic sites in blebbistatin or CCE-treated cells (derived from the mean of 8 wells per experiment) was normalised to the proportion of large sites in control samples, per experiment (N=3 independent experiments). Statistical significance was assessed with 2-way ANOVA sidak’s multiple comparison test , ** P<0.05 and *** P<0.005.


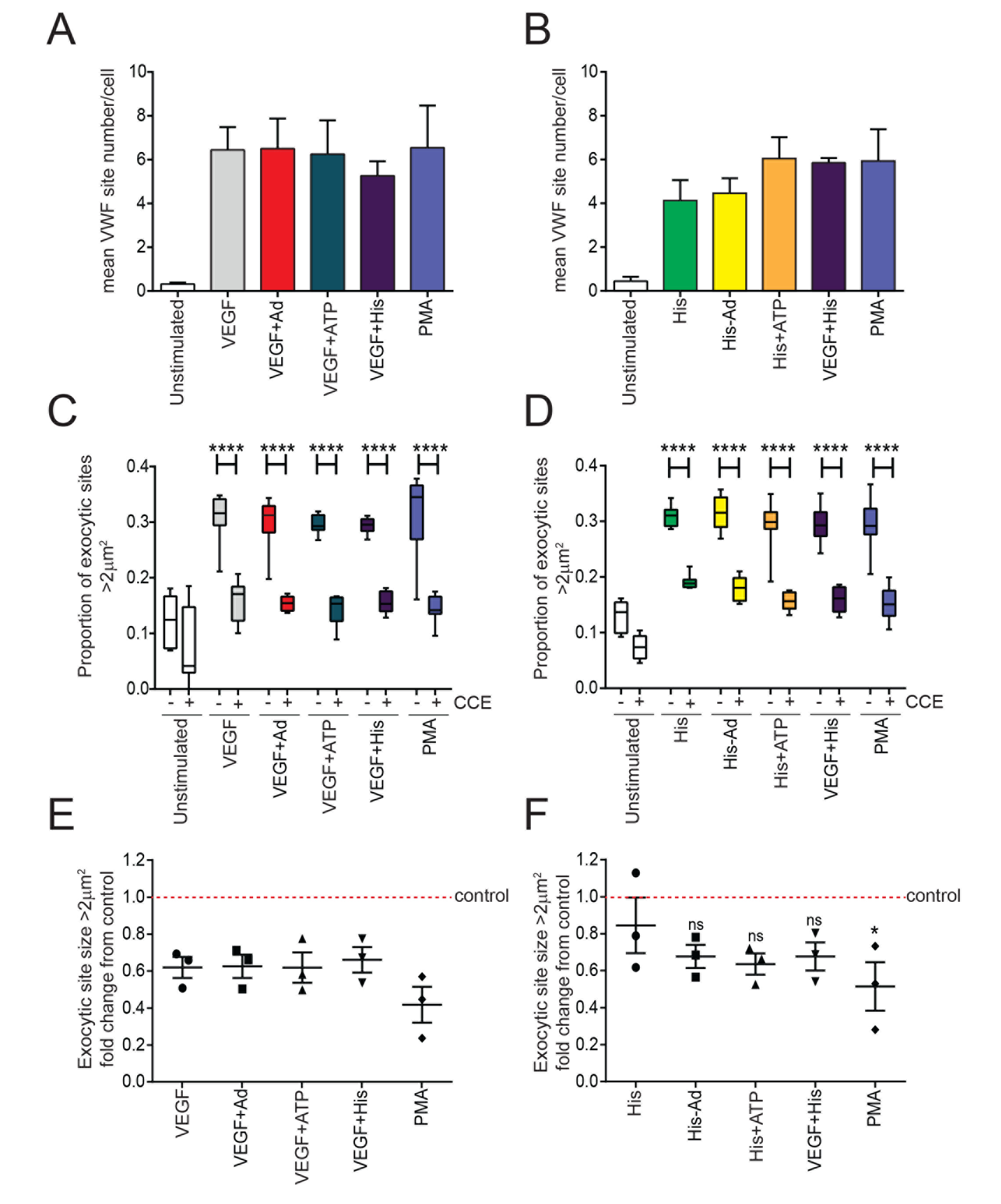
Figure S3. **Analysis of actin ring function with a variety of secretagogues.**

HUVECs were treated with or without 1µM CCE before being stimulated with either 40ng/ml VEGF alone or in combination (A, C, E), 100µM histamine alone or in combination (B, D, F) or 100ng/μl PMA (A-F) for 10 minutes, followed by staining for external VWF and the nucleus. Images were acquired using the Opera high-content screening confocal microscope. Nine fields of view were acquired per well, and eight wells imaged per condition. Representative experiments are shown (A-D) from N=3 (A, C) and N=4 (B, D) independent experiments. (A-B) Mean number of exit sites per cell per well with the different secretagogue combinations. Bars represent SEM. (N=8 wells). (C-D) The proportion of exocytic sites greater than 2µm^2^ following stimulation with secretagogues alone or in combination and with or without CCE (1µM). Boxes represent 25th-75th percentiles, whiskers represent minimum and maximum values. (N=8 wells). (E-F) The mean proportion of exocytic sites with area greater than 2µm^2^ following stimulation with a number of secretagogues in the presence of CCE normalised to the mean proportion of large sites in control samples. Bars represent SEM. Mean value is derived from the mean of N=8 wells per experiment (E; N=3, F; N=4). Statistical significance was assessed using 2-way ANOVA with Sidak’s multiple comparison test (C-D) or 1-way ANOVA with Dunnet’s multiple comparisons test (E-F). * P<0.05, **** P<0.0001, ns=not significant.


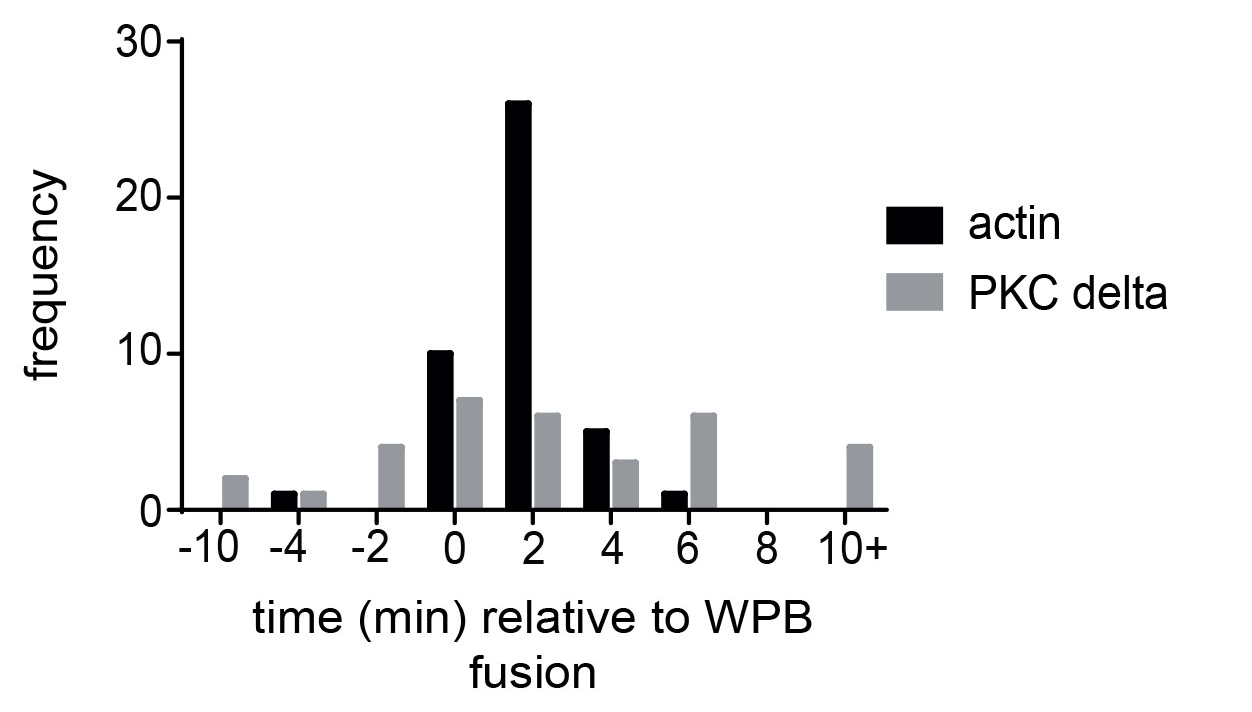


Figure S4 **The timing of PKC delta recruitment to exocytic sites**

HUVECs were nucleofected with mcherry-Pselectin.lum and PKCδGFP or mcherry-Pselectin.lum and lifeact GFP and imaged with a spinning-disk confocal microscope in the presence of 100 ng/ml PMA (n=9). Z stacks were acquired at a spacing of 0.5 µm every 5 s for 10 min. The timing of actin and PKCδ recruitment was plotted relative to the point of fusion (as determined by the loss of mcherry-Pselectin.lum).


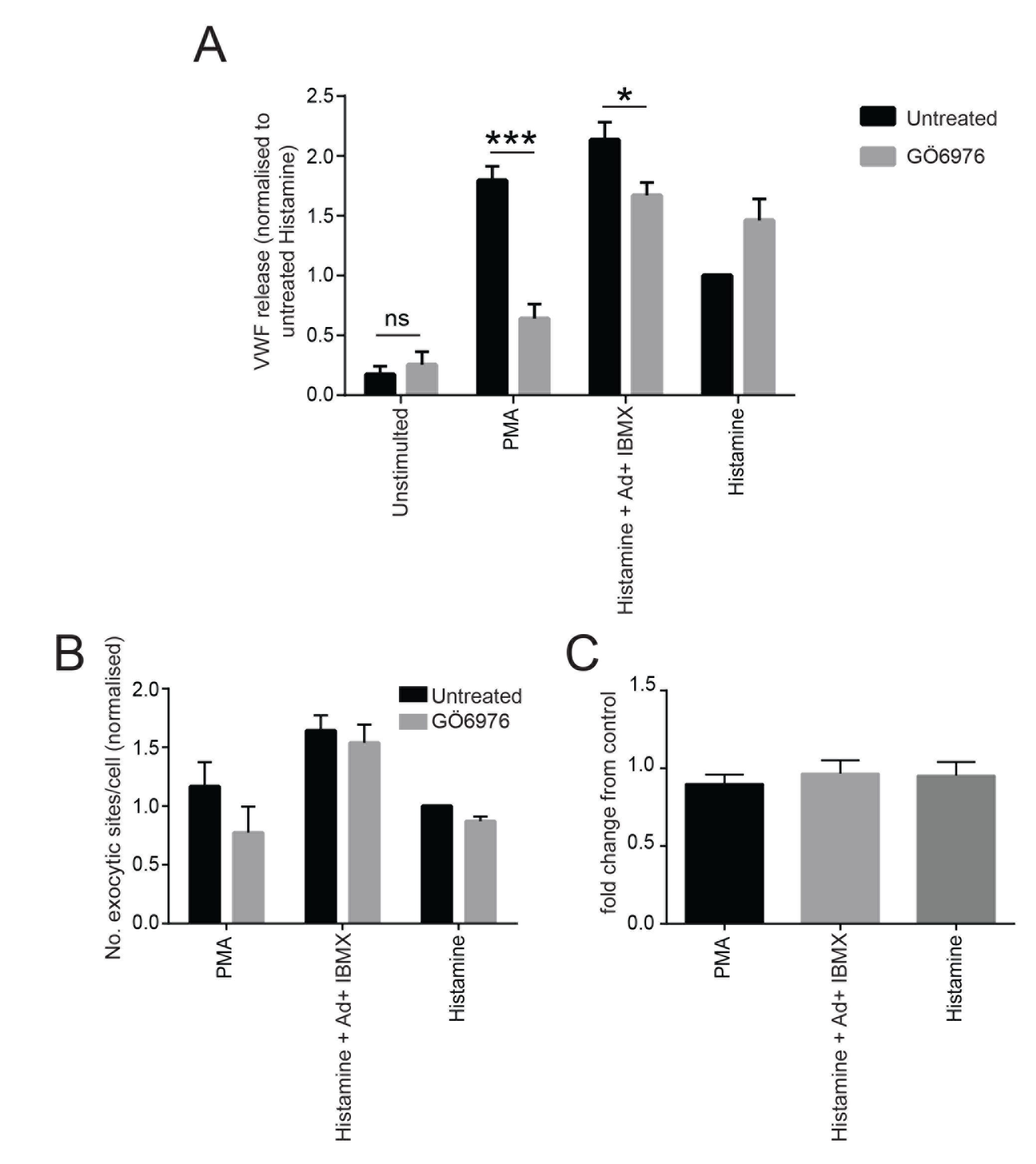
Figure S5. **Effect of GÖ6976 on exit site size and number.**

HUVECs were treated with or without 1µM GÖ6976 for 15minutes before being stimulated with 100ng/μl PMA, 100µM Histamine or 100µM Histamine/10µM adrenalin/100µM IBMX for 10-30 minutes. The VWF secretion was determined by ELISA (30 minutes stimulation) (A) or following 10 minutes stimulation, samples were stained for external VWF, wheat germ agglutinin (WGA) to label the plasma membrane with or DAPI to label the nucleus. Images were acquired using the Opera high-content screening (PerkinElmer) confocal microscope. Nine fields of view were acquired per well, and eight wells imaged per condition. Data from a representative experiment is shown (n=4) (B,C). (A) The amount of VWF released is determined relative to cells stimulated with Histamine (B) Mean number of exit sites/cell with the different secretagogues in the presence or absence of GÖ6976. (C) The proportion of exit sites greater than 2µm^2^ with different secretagogues in the presence of GÖ6976 normalised to an untreated control.
